# Palliative and end-of-life care in COVID-19 management in sub-Saharan Africa: a matter of concern

**DOI:** 10.11604/pamj.supp.2020.35.25288

**Published:** 2020-08-03

**Authors:** Marie Josiane Ntsama Essomba, Laura Ciaffi, Paul Owono Etoundi, Agnès Esiene

**Affiliations:** 1 Department of Internal Medicine and specialties, Faculty of Medicine and Biomedical Sciences, University of Yaoundé I, Yaoundé, Cameroon,; 2 Geriatric Unit, Yaoundé Central Hospital, Yaoundé, Cameroon,; 3 Unité Mixte Internationale TransVIHMI, UMI 233 IRD-U1175 INSERM, Université de Montpellier, Montpellier, France,; 4 Department of Emergency medicine, Anaesthesiology and Critical care, Faculty of Medicine and Biomedical Sciences, University of Yaoundé I, Yaoundé, Cameroon,; 5 Department of Emergency medicine, Anaesthesiology and critical care, Yaounde Central Hospital, Yaoundé, Cameroon

**Keywords:** COVID-19, palliative care, sub-Saharan Africa

## Abstract

The COVID-19 pandemic has strained health care systems beyond capacity resulting in many people not having access to life-sustaining measures even in well-resourced countries. Palliative and end-of-life care are therefore essential to alleviate suffering and ensure a continuum of care for patients unlikely to survive. This is challenging in sub-Saharan Africa where lack of trained teams on basic palliative care and reduced access to opioids limit implementation of palliative and end-of-life care. At the same time, health care providers have to cope with local cultural conceptions of death and absence of advance care directives.

## Commentary

COVID-19 first emerged in China in December 2019 and spread rapidly throughout the world. Since the World Health Organization (WHO) statement on March 11, 2020, COVID-19 outbreak has turned into a pandemic with 16,795,762 people infected at July 27, 2020 [1]. The disease has brought a lot of suffering and continues to generate huge losses for families and communities. As we are writing this paper, 660,667 people succumbed worldwide to the disease [1]. Despite better understanding of pathophysiological mechanisms and several studies published, no curative treatment nor vaccine have yet been found against COVID-19. Although critical care is recognized as a pillar in management of COVID-19, some critically ill patients have been denied life-sustaining therapy [2] due to the lack of beds in overburdened hospitals. Thus, access to palliative care has become essential in comprehensive COVID-19 management: to address the medical needs of patients who will not recover from SARS-COV-2 infection but also to respond to the physical, psychological and spiritual needs of patients and their families [3-5]. Therefore WHO recommends that palliative care interventions should be made accessible at each institution that provides care for COVID-19 patients [6].

In this COVID-19 era, we cannot refrain from having concerns about sub-Saharan Africa (SSA) countries where health systems are ill-prepared to meet the needs of too many critically ill patients. WHO gives clear recommendations for the integration of basic palliative care, including relief of dyspnea or other symptoms and social support by all doctors, nurses, social workers and others caring for persons affected by COVID-19 [6]. But are all these personnel trained in the practice of palliative care? What about the accessibility of the list of drugs proposed by WHO for COVID-19 palliative care, especially morphine? What is the availability of adapted equipment for those patients? In addition, there is still a major gap in the approach to palliative and end of life care to be tackled by health workers in SSA countries. The majority of SSA countries have developed strategies to manage infected patients but few consider end stage disease and therefore do not propose palliative care in their guidelines. Indeed, patients with severe COVID-19 who are unable to access intensive care in resource-limited settings will probably suffer and die at home or in healthcare facilities without any palliative approach. In fact, if palliative care is a developed discipline in high-income countries, it remains neglected in SSA [7]. COVID-19 epidemic has come just to underline the limits of critical care in SSA countries and to remind to the medical community the importance of incorporating palliative care in COVID-19 management. Unfortunately, the medical community as well as the health system in general is totally unprepared for this challenge. In high resources countries, COVID-19 mortality has been very high among the elderly (median age of 21,551 dead patients in Italy was 80) [8], an age group in which the practice of advance care plan is more widely accepted and practiced. In our context, geriatric medicine is still at its beginning and the integration of discussion about desire of life support is not a standard with elderly patients even less with all the other age groups.

For most people in SSA, talking about end of life and death is considered a taboo. Many African societies do not recognize advance care directives and are not prone to refuse or discontinue life-sustaining treatment [9]. Hence, expectations of patients and families when coming to hospital are to preserve life by any means, even in case of hopeless situations. The rapidly changing evolution of COVID-19 can lead to inaccurate prognosis, making it difficult to prepare patients and their families to probable death and causing alongside psychosocial suffering. Restrictions of social interactions as well as stigma around COVID-19 patients and the distress of their close ones must be taken into account. A well conducted and honest discussion with patients unlikely to survive and their families is very important but rarely done. Current training of medical doctors and nurses on management of COVID-19 in SSA is focused on preventing and treating the disease, palliative and end of life care are not included in the program. There is a lack of interest about dying patients and the extremely important needs that these can have. Health care providers must be trained on managing particular issues associated with dying COVID-19 patients. Availability of medications is essential for implementing palliative care. Clinicians are not trained on how to manage uncontrolled breathlessness or pain.Opioids medications are not widely available in SSA and lack of clear policy on opioids use often leads to fear of misuse and abuse. Therefore, many COVID-19 patients with respiratory failure will not benefit from opioids as they are not considered a priority [10]. While this health crisis continues to impact the continent, governments prioritize resources to provide access to disease-specific treatments and personal protective equipment (PPE) while neglecting all those patients who will not recover and die in suffering.
